# Causes and Treatment of Chronic Middle Ear Catarrh

**Published:** 1894-12

**Authors:** R. E. Moss

**Affiliations:** San Antonio, Texas


### TEXAS MEDICAL JOURNAL.

ESTABLISHED JULY, 1885.

PUBLISHED MONTHLY.—SUBSCRIPTION $2.00 A YEAR.

VOL. X. AUSTIN, DECEMBER, 1894. NO. 6.

### Original Contributions.

For Texas Medical Journal.

### CAUSES AND TREATMENT OF CHRONIC MIDDLE EAR CATARRH.

BY R. E. MOSS, M. D., SAN ANTONIO, TEXAS.

[Read at annual meeting of Southwestern Texas Medical Society, Oct., 1894.]

THIS disease is by far the most common cause of partial or total loss of hearing, and as the hearing is only second in importance to the sight, we readily understand that it is a subject of great importance. I will first briefly allude to the anatomy of the parts primarily involved, and the effects secondarily caused by this affection. The middle ear might be styled the transmitting apparatus, and contains the three bones, malleus, inchus and stapes. The malleus with its long arm attached to the membrana tympani, short arm or process to superior posterior wall, and its articulating surface with the inchus. The inchus has its short process attached to wall above, and its long arm acts a lever and articulates at the extremity with the stapes, which fits into the round window. The inchus and malleus articulate with each other by a half ball and socket movement. This is a most marvelous piece of mechanism, and not only transmits the sound waves, but greatly increases them when these joint surfaces work in a harmonious manner. The space above these is called the attic, which opens into the mastoid antrum. We have the Eustachian tube connecting this drum or middle ear space with the nasopharynx. All of this space is lined with delicate mucous membrane, which is continuous with that of the nasopharynx. Catarrh of the middle ear is not strictly a local process, but is one of the many manifestations of a general inflammatory tendency of the mucous surfaces, of which the Eustachian tubes, tympanum and mastoid antrum are the parts directly involved in this disease.

We find the nose, nasopharynx, fauces and larynx affected, or evidences showing that they have been. Behind all of this local trouble, there must be a general cause, as a rule, and whether we speak of it as an inherited diathesis, or general malnutrition, we must admit its importance upon the duration and treatment of this affection. Any systematic condition which lowers the vitality of the individual, enfeebles the mucous membranes, rendering them less able to resist agents that act as exciting causes; therefore any condition which produces a permanent lowering of the vitality of the body, acts as a predisposing cause. This will include a great variety of diseased conditions and environments.

Heredity has always been classed as one of the most frequent predisposing causes of this trouble. It is easy to understand how an impaired vitality may be transmitted from parent to child, and in connection with similar environments, such as climatic and hygenic conditions, they would be more susceptible to catarrhal diseases. But the transmission from parent to child of a specific tendency or diseased condition is rarely if ever the case, unless it be in syphilitic and scrofulitic conditions. Probably the great majority of cases begin at or before puberty, therefore age would be classed as predisposing. The disease is such an insidious one, and never gets well of itself, or spontaneously, so those who have it once, have it always. The great majority of patients are not aware of any trouble until some acute exacerbation from exposure to climatic influences, or troublesome tinnitus, causes them to consult a physician. It is quite common, when making a routine office examination, to find an opaque retracted m. t., and inform the patient that he has a chronic ear trouble, much to his surprise.

Climatic conditions, of course, are potent factors in causing this condition, and generally speaking, damp, sudden changes, like we have in the early spring months in the States north and east, favor acute attacks, and exacerbations of chronic ones. To enter into detail in regard to how these changes in temperature, barometric conditions of the atmosphere, etc., bring about diseased conditions of the mucous surfaces, would prolong this paper beyond reasonable length. Modern style of living, with all of its boasted conveniences, comforts, and elegancies, is largely instrumental in preparing the system to take on catarrhal congestions or inflammations. Dyspepsia, in all of its different forms, is a frequent cause, particularly the fermentative variety with acid eructations. Diet, therefore, is of utmost importance, especially in children, not only as a cause but from a standpoint of treatment. It is utterly impossible to cure these cases where there is much disturbance of the digestive organs, without first improving the assimilative functions of the body. Malnutrition causing enfeeblement of the circulation, with nervous stasis, local congestions and relaxation of mucous membranes, is responsible for many cases, and markedly aggravates a case already established. Long hours of work, with professional men, business men, clerks, students, etc., lowering the vitality of the individual, thereby decreasing the resisting power to atmospheric changes. The housewife, with her numerous cares, the society woman, with the jealousies, rivalries, and the physical and mental strain imposed upon them by fashionable life with its late hours, heated rooms, unwholsome, late suppers, etc., adds many patients to the list. This drain upon the nervous energy further complicates matters by enfeebling the functions of all parts of body, thereby causing conditions which are strictly modern, and often alluded to as neurasthenia, or nervous prostration. All of these influences predispose to catarrh, not only of the ear, but of all the air tract.

As exciting causes, we have repeated attacks of cold, climatic changes, excesses of all kinds in diet, alcohol, tobacco, etc. An otitis media may have existed for a long time unnoticed, when sudden changes in temperature, or imprudence, will cause an exacerbation, and the patient attribute all of his trouble to that attack, when in reality his otitis may have existed for years. Occlusion of nares, deviation of septum, closure of Eustachian tubes, either from inflammation or pressure of adenoids, and pharyngeal tonsil.

The inflammation of a nasopharyngitis may extend along the tube to the middle ear, or by partial or complete closure cause rarefaction of the air in the middle ear, thereby causing congestion of the lining membrane, which, if long continued, brings about pathological changes. Organic heart troubles, especially valvular insufficiency, cerebral disease, stomach troubles, menstrual disorders, dental caries, etc., are all put down as causes.

The pathological changes in otitis media may be divided into two classes, the hypertrophic, and the atrophic, or sclerotic. The atrophic changes usually follow the hypertrophic, however, and is really a continuation of the same process. The primary condition may be inflammatory, or mechanical, from occlusion of the Eustachian tube. This latter condition causes an exudation of serum, which may become absorbed, leaving little damage, or marked hyperæmia may set in, with plastic exudate infiltration of tissues, cell proliferation and true hyperplasia. As a result of long standing hyperæmia or inflammation, there is an exudate, especially in exacerbations, composed of serum, round cells and exfoliated epithelium. The serum is gradually absorbed, whilst the round cells increase both in size and numbers, forming bands of lymph which become connective tissue. There is also marked thickening of the m. t., and the entire lining of the middle ear. The tendency of this new formed tissue is like cicatricial tissue elsewhere, and that is to atrophy and contract, causing retraction of the m. t. with partial or complete immobility of the ossicles and increased pressure of the semicircular canals.

It is during this stage of hyperæmia, and before atrophy begins, that treatment is of any avail. This stage of atrophy progresses until the nutrition of the parts becomes interfered with to a marked degree, then the mucous membrane assumes a white appearance, getting quite thin, and calcareous deposits occur at this time, as noticed by a white line encircling the outer border of the m. t. The ligaments of the bones also receive these deposits, until finally we have complete anchylosis. The inflammation even extends to the periostrum of the ossicular chain, therefore we may have anchylosis, partial or complete, due to fibrous, calcareous or osseous deposits.

The labyrinth becomes involved secondarily in these cases of long standing otitis due to increased pressure upon the fenestra oralis, causing congestion of the labyrinthine blood vessels and acoustic nerve. Therefore we can readily see how essential the operation for removal of the drum membrane and ossicles, thereby preventing further changes of the acoustic nerve. If the operation is too long delayed, we have the retrograde process set up in this nerve which follows prolonged congestion of a nerve, namely atrophy, and finally complete loss of function. Since anatomists in recent years have discovered a decussation of nerve fibres from one ear to the other, and from one nucleus to the other, we see how necessary, and I might say, imperative it is, when finding one ear in this condition, and the other only slightly affected, or not at all, to remove the ossicles, thereby taking off the pressure, and preventing secondary involvement of the other ear, not by sympathy, but directly affecting the nerve fibres which cross over to the opposite ear.

The symptoms of catarrhal otitis may be so slight as not to be noticed until the patient's attention is called to it by some one who notices the dullness of hearing. Then again, the symptoms may be very distressing, as when there is marked tinnitus or closure of Eustachian tube, causing patient's voice to sound so far away, or as if muffled. Deafness of course is the most pronounced symptom, and varies very greatly. Some patients even with pronounced catarrhal otitis retain their hearing to a useful degree throughout life, whilst others become totally deaf at an early age. There are other symptoms, such as otalgia, neuralgia hallucinations, vertigo, etc., but they are usually transitory, yet at times very distressing. In regard to prognosis there is little to be said, and yet our patients all want to be told positively in regard to their condition, and whether they can be cured. There are so many conditions to be considered before forming an opinion. The general health of the patient, predisposition to catarrhal processes, conditions present in nose, nasopharynx, Eustachian tubes, appearance of m. t., whether retracted or not, mobility of ossicles, duration of trouble, age of patient, etc., etc. To take up each of these subjects would consume too much time; suffice it to say we should promise little in any case, and be very guarded in giving much encouragement. Yet in recent cases where the middle ear catarrh is caused by obstructions, and there are no pronounced secondary changes, the results are frequently very brilliant. If we find the tubes patent, m. t. retracted, thickened or sclerosed, slight mobility of ossicles, constant tinnitus or vertigo, the prognosis is most unfavorable. This condition is gradually intensified with or without treatment, unless we remove the ossicles, until the patient gives up all treatment in despair, and uses the hand behind the ear, or gets some one of the many patent devices advertised to cure or improve the hearing.

The treatment of this disease is constitutional, local, and removing the cause or causes. Our first duty to the patient is to have him overhauled, so to speak, by his family physician, if we have reason to believe any of the bodily functions are not performed in a normal manner. The patient should have specific instructions in regard to a cold sponge, shower, or tub bath every morning, how and when to exercise, proper mode of dress, how to overcame constipation by diet, exercise, massage and habit. This part of the treatment is very important, and the patient must be impressed with the benefit to be derived from faithfully observing in detail the advice given. The nose comes in for a large share of attention. Spurs, exostosis and deflected septums must be removed and straightened. I have never seen a single case where there was a large spur or deflected septum that there was not an otitis media. Hypertrophy of turbinated tissues must be cured, especially posterior hypertrophies of inferior bone. The nosopharynx must receive careful attention, and all adenoid or lymphoid tissue promptly and thoroughly removed, preferably with a Gruber curette, the patient fully under an anæsthetic. The accessory cavities and especially the ethmoid cells should receive careful attention. An astringent alkaline spray, used hot to nose and nasopharynx, is of some value, but upon the whole disappointing to both patient and physician. The Eustachian tube should be carefully looked after, if closed, or its lining membrane inflamed. In acute exacerbations we should be very careful about rough treatment or trying to open by forced inflation. It is better to rely upon general treatment, removing all nasal obstructions, using hot irrigations to nasopharynx, and topical applications to mouth of tubes, with nitrate of silver gr. xl.–℥. Later it is well to inflate by means of catheter and douch apparatus, containing iodine, camphor, menthol, eucalyptol or whatever the one in charge may prefer. I find where there is beginning retraction of m. t., and immobility, that Seigle's pneumatic speculum, properly and persistently used, is of very great value. There are many adjuncts to and changes in treatment that each individual practitioner must select or adopt in each case treated. After faithful treatment for three or four months where the watch is only heard a few inches, or where there is anchylosis of ossicles or sclerosis, we should advise and insist upon prompt removal of m. t. and ossicles. This operation unfortunately, like all surgical procedures, is not always successful, yet in suitable cases, properly diagnosed, the results are highly satisfactory. In this, like all other surgical operations, we should first know how to make a correct diagnosis, excluding disease of labyrinth, or acoustic nerve, and do not postpone too long, or we will have secondary labyrinthine changes.

